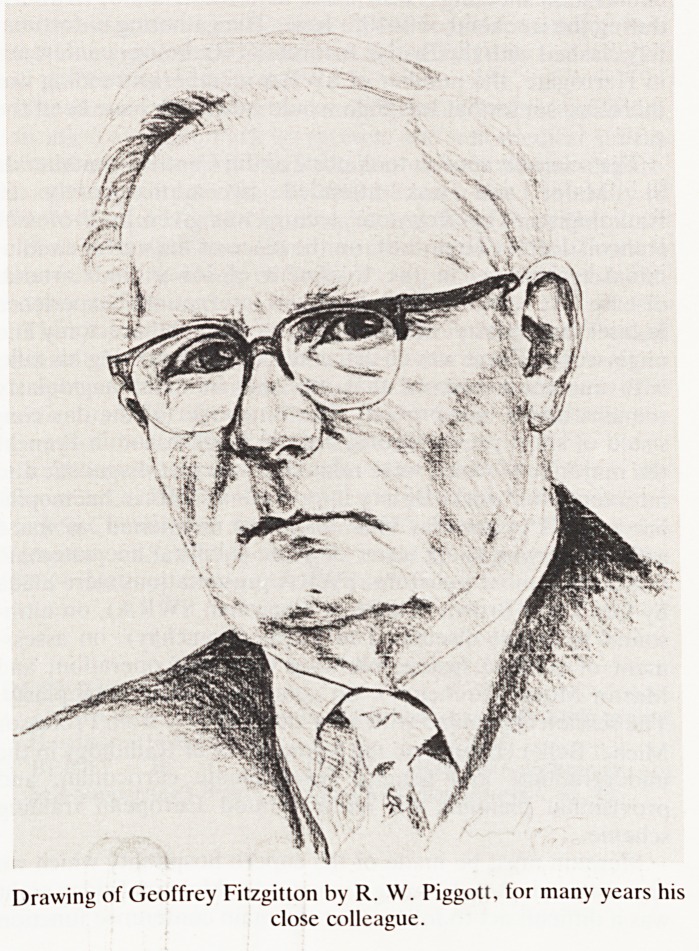# Geoffrey Molyneux Fitzgibbon

**Published:** 1990-09

**Authors:** 


### OBITUARY

### Geoffrey Molyneux Fitzgibbon. MD., FRCS.

Geoffrey Fitzgibbon, best known as Fitz, was born on 25th August 1903 and died on the 25th May 1990. He received his early surgical training at St Thomas's under Philip Mitchener obtaining his Fellowship and an M.D. in Midwifery and Gynaecology by the age of 26.

At this time he and a friend were looking for more senior posts. They agreed to decide by the toss of a coin which of them should apply for a vacancy at Bristol General Hospital and so it was that Fitz came to Bristol and was appointed Senior Resident Officer, a post comparable to Senior Registrar today but with wider responsibilities. At B.G.H. he worked under Ernest Hey-Groves and he always acknowledged the influence of his two great surgical teachers.

In 1932 when he was 29 two vacancies occurred on the staff of B.G.H., one in Midwifery and Gynaecology and the other in General Surgery. It is remarkable that the appointment committee offered him the choice of the two posts. He selected that of Assistant Honorary General Surgeon. In those days before the war the term General Surgeon meant that, a field now covered by twelve separate surgical specialities.

It was at the B.G.H. that he met Priscilla Harrison, then House Physician to Dr Carey Coombes. They were married in 1933 and so began a happy partnership which was to last for 57 years.

When the Bristol Royal infirmary and General Hospital were amalgamated in 1940 he moved to the B.R.I. and played a full part in the management of the Bristol air raid casualties.

In 1942 he joined the R.A.M.C. and was seconded to Sir Harold Gillies' Unit at Park Prewitt Hospital, Basingstoke to train in Plastic Surgery. He took naturally to this new discipline. Innovative and a decisive operator he quickly mastered the delicate handling required for the various grafting procedure. His clear mind enabled him to plan multi-stage operations and to visualise the end results weeks or months later.

It was there he was given the responsibility of selecting and assembling the team for No. 5 Maxillo-facial and Surgical Unit and subsequently taking it to join the British Expeditionary Force serving first in France and then in Belgium and Germany. He was a natural leader managing his team not by discipline but by setting an example and trusting others as others always trusted him.

He was at his best when the odds were loaded against him. To him problems were challenges to be faced, enjoyed and brought to a successful conclusion. Always cheerful, modest and good humoured, in control of the situation, never fussing and never cross, he was the ideal man to work with, treating all staff regardless of rank with the same consideration. He was resourceful, as when on the battle front a vital piece of equipment, the suction apparatus broke down, he commandeered an army truck and used the air inlet of the engine. He was at home in a tented hospital as in a purpose built operating theatre.

After victory in Europe he was sent to India and was demobilised in February 1946 with the rank of Lieutenant Colonel. On leaving the army he returned as General Surgeon to the B.R.I. where his interest in teaching was reflected in his appointment as the first clinical dean in that year.

Among the last of the true General Surgeons he was one of the first of the new generation of Plastic Surgeons. He was quick to recognise the role Plastic Surgery could play in the treatment of late cancer. Many patients had developed extensive disease untreatable by surgery of the day or by more radiotherapy. Fitz with his wide background experience knew he could remove the disease, then repair the defects by using the skills he had acquired in repairing large tissue losses due to gun shot wounds and which the major advances in anaesthesia and control of infection by penicillin had made so much safer. So it was that he was able to offer these previously hopeless patients a chance of a future, no problem seemed too big for him. His pioneer work in this field first at B.R.I. and later at Frenchay established for him a national and international reputation. It took over ten years for the management of cancer to become generally accepted as a routine procedure and written into the requirements for training of Plastic Surgeons. It is surprising today that this enterprise was resisted by the pioneers of those days. Many patients are in debt to him and only 10 days before he died a farmer from Cornwall telephoned him on the 40th anniversary of the operation to remove a massive fibrosarcoma and repair of the wound.

His greatest achievement was setting up the Dept of Plastic Surgery at Frenchay in 1949, the hospital built as a wartime emergency one and used by the Americans for their 295 General Hospital. After lying empty it was graduallly being brought back into use as a Regional Centre for Neuro, Thoracic and Plastic Surgery. He set up the unit with an enduring spirit of friendship and comradeship amongst all

Drawing of Geoffrey Fitzgitton by R. W. Piggott, for many years his close colleague.

who worked there establishing an openness and team spirit. In the early days he gave Frenchay a sense of direction, and started the Medical Library. He treated everyone, whether colleague, junior staff, nurse secretary or theatre assistant with the same respect often asking for their opinions. Innovative and quick to adopt new ideas he made the Department of Plastic Surgery so sought after that it was rarely necessary to advertise vacancies, to Frenchay came trainees from all parts of the world.

His wide interests covered the arts, travel and history but he was never happier than when with his totaally united family. He loved constructing his beautiful gardens and enjoying his collection of pictures. He of course owes much to the devoted and constant support given by Priscilla and with her he regularly entertained visiting surgeons and friends until illness intervened. It was typical of him that in the late fifties they had a party at their home in Wickwar to share their experiences after visiting Egypt and he asked not just his colleagues but everyone who worked in the department.

He was a founder member of the British Association of Plastic Surgeons and its President in 1966. As President of the Bristol-Medico Chirurgical Society in 1965 he chose "Medicine and the Arts" as the subject for his Presidential Address. He enjoyed his research for this travelling the country in quest of pictures and sculptures with a medical interest. As Gillies Memorial Lecturer in 1967 his subject was "The Commandments of Gillies".

It is a tribute to him, that up to now Frenchay has produced four Presidents of the British Association of Plastic Surgeons, four National Meetings have been held in Bristol and a ward in the new Frenchay Hospital is to be named after him. The Frenchay spirit has been enduring and typified the 40th Birthday celebrations of the Department held in 1989 when he was guest of honour.

After retirement he was Chairman of the North Nibley Parish Council and he also helped local General practitioners taking on holiday duties for many years when he earned the respect of patients by his willingness to listen at length to their problems and he dispensed wisdom more often than drugs.

He bore his terminal illness and its problems with fortitude.

He is survived by his wife, four daughters, a son and 13 grandchildren.

## Figures and Tables

**Figure f1:**